# Visual feedback-dependent modulation of arousal, postural control, and muscle stretch reflexes assessed in real and virtual environments

**DOI:** 10.3389/fnhum.2023.1128548

**Published:** 2023-04-04

**Authors:** Daniel D. Hodgson, Jordan A. King, Osman Darici, Brian H. Dalton, Taylor W. Cleworth, Tyler Cluff, Ryan M. Peters

**Affiliations:** ^1^Faculty of Kinesiology, University of Calgary, Calgary, AB, Canada; ^2^Biomedical Engineering, University of Calgary, Calgary, AB, Canada; ^3^School of Health and Exercise Sciences, University of British Columbia Okanagan, Kelowna, BC, Canada; ^4^Faculty of Health, York University, Toronto, ON, Canada; ^5^Hotchkiss Brain Institute, University of Calgary, Calgary, AB, Canada

**Keywords:** muscle stretch reflexes, virtual reality, electromyography, electrodermal activity, H-reflexes, tendon vibration, postural sway

## Abstract

**Introduction:**

The mechanisms regulating neuromuscular control of standing balance can be influenced by visual sensory feedback and arousal. Virtual reality (VR) is a cutting-edge tool for probing the neural control of balance and its dependence on visual feedback, but whether VR induces neuromodulation akin to that seen in real environments (eyes open vs. closed or ground level vs. height platform) remains unclear.

**Methods:**

Here we monitored 20 healthy young adults (mean age 23.3 ± 3.2 years; 10 females) during four conditions of quiet standing. Two real world conditions (eyes open and eyes closed; REO and REC) preceded two eyes-open virtual ‘*low*’ (ground level; VRL) and ‘*high*’ (14 m height platform; VRH) conditions. We measured arousal via electrodermal activity and psychosocial questionnaires rating perceived fear and anxiety. We recorded surface electromyography over the right soleus, medial gastrocnemius, and tibialis anterior, and performed force plate posturography. As a proxy for modulations in neural control, we assessed lower limb reflexive muscle responses evoked by tendon vibration and electrical stimulation.

**Results:**

Physiological and perceptual indicators of fear and anxiety increased in the VRH condition. Background soleus muscle activation was not different across conditions; however, significant increases in muscle activity were observed for medial gastrocnemius and tibialis anterior in VRH relative to REO. The mean power frequency of postural sway also increased in the VRH condition relative to REO. Finally, with a fixed stimulus level across conditions, mechanically evoked reflexes remained constant, while H-reflex amplitudes decreased in strength within virtual reality.

**Discussion:**

Notably, H-reflexes were lower in the VRL condition than REO, suggesting that these ostensibly similar visual environments produce different states of reflexive balance control. In summary, we provide novel evidence that VR can be used to modulate upright postural control, but caution that standing balance in analogous real and virtual environments may involve different neural control states.

## 1. Introduction

Human standing balance is maintained through a dynamic integration of multiple sensory and motor systems. Alpha-motor neurons (αMNs) are the final common pathway for motor control, with their output being the culmination of complex spinal and supra-spinal integration processes (Burke et al., 1983; Pierrot-Deseilligny and Burke, 2005). Interestingly, the neural control mechanism that support standing balance do not appear to be fixed; rather, they readily adapt to changes in static posture, like standing vs. sitting (Hayashi et al., 1992; Cattagni et al., 2014), as well as different motor behaviors, like standing vs. walking (Capaday and Stein, 1986). The neural control of standing appears to be further modulated in the presence of different task demands and postural threat, such as when there is a chance of being perturbed (Horslen et al., 2013; Lim et al., 2017), when the eyes are closed (Roman-Liu, 2018), or when standing at the edge of a height platform in real (Carpenter et al., 1999, 2004; Sibley et al., 2007; Horslen et al., 2013, 2014, 2017, 2018; Naranjo et al., 2015, 2016) and virtual environments (Cleworth et al., 2012, 2016; Nielsen et al., 2022).

Here we follow the general approach of using muscle stretch reflex excitability to characterize the neural mechanisms supporting standing balance (e.g., Davis et al., 2011; Horslen et al., 2013, 2017, 2018). Our focus is on lower-limb muscle stretch reflexes as a proxy for modulations in the neural control of standing. In response to changes in muscle length, spindle afferents generate reflexive activation through the myotatic stretch reflex arc (Liddell and Sherrington, 1924, 1925; Matthews, 1991). Muscle spindles are also unique in that they have their own dedicated motor innervation (‘*fusimotor neurons*’ or 𝝲MNs), enabling active control over receptor sensitivity by the central nervous system (Hulliger, 1984; Burke, 2021; Dimitriou, 2021). Context-dependent adaptations of the stretch reflex may also result from altered descending commands that modulate the excitability of spinal interneurons and Renshaw cells, and/or changes in muscle spindle sensitivity via 𝝲MN activation (Burke et al., 1983; Hulliger, 1984; Dimitriou and Edin, 2010; Burke, 2021; Dimitriou, 2021, 2022). Furthermore, muscle stretch reflexes are traditionally assessed in two different ways, which offer complementary bits of information: mechanical stimulation (imposed joint movements, tendon taps, and muscle/tendon vibration) and electrical nerve stimulation (Hoffman’s reflex). The major distinction between mechanical and electrical stimulation is that the former relies on mechanotransduction by the muscle spindle, whereas the latter bypasses these mechanoreceptors and directly activates the peripheral nerve along its length. This is important because changes in muscle spindle sensitivity via 𝝲MN input may only be inferred using mechanical stimuli, whereas electrical stimuli give an indirect measure of spinal excitability and presynaptic inhibition (Pierrot-Deseilligny and Burke, 2005; Horslen et al., 2013, 2018).

In real-world environments, muscle stretch reflex excitability appears to be modulated in the presence of increased postural demands, altered visual feedback, and postural threat. For instance, Hoffman (H) reflexes in the soleus were reduced during standing compared to sitting, even when background αMN activity was matched in the seated position (Cattagni et al., 2014). Postural control is challenged when the eyes are closed (Roman-Liu, 2018), however, H-reflexes appear to not be significantly affected by closing the eyes (Sibley et al., 2007). Furthermore, Sibley et al. (2007) demonstrated that H-reflexes are reduced in amplitude when standing on the edge of a real height platform, which was taken as evidence of altered pre-synaptic inhibition or fusimotor drive. By comparing T- and H-reflexes during sympathetic arousal in the same participants, researchers have suggested the presence of enhanced muscle spindle sensitivity under threatening conditions (Davis et al., 2011). Horslen et al. (2013), found that muscle stretch reflexes evoked with tendon taps become sensitized in response to postural threat from a height platform, while the excitability of the spinal circuitry – assessed with H-reflexes – remained largely unaffected. Additional studies have suggested that H-reflexes reduce in amplitude under enhanced postural threat (Llewellyn et al., 1990; McIlroy et al., 2003; Nafati et al., 2004; Sibley et al., 2007), rather than remain unchanged (Horslen et al., 2013). To our knowledge, no studies have investigated such effects within virtual environments and how this compares to simply removing vision by closing the eyes, which is a main objective of the present study. Horlings et al. (2009) induced changes in postural control during quiet standing in both eyes closed and virtual reality (VR) low height conditions compared to eyes open, however, muscle activity and reflex responses were not monitored.

Virtual reality has emerged as an increasingly popular tool for studying human motor control. As a validation of VR, balance control and physiological and/or psychological stress appear to occur similarly in real and VR environments (Meehan et al., 2002; Cleworth et al., 2012, 2016; Nielsen et al., 2022). For example, Cleworth et al. (2012) compared responses to standing on an elevated platform vs. at virtual height in an environment matched in scale and visual detail. The authors reported similar changes in the amplitude and frequency of centre of pressure (COP) excursions, electrodermal activity (EDA), anxiety, fear, and balance confidence for real and VR height conditions, providing evidence for the potential utility of VR as a tool for manipulating balance control and arousal. The safety and practical challenges (cost and space requirements) of having participants at the edge of a real height platform, building, or cliff is a barrier to studies of this sort. VR may offer a means of circumventing these challenges by simulating tasks and environments that create threat and challenge the balance system. Commercial VR systems (e.g., Oculus Rift, HTC Vive, etc.) have decreased in cost while providing better quality visual feedback in the last 5-10 years. Although VR may offer a fruitful tool to explore how the balance system adapts control for a wide range of visual environments, it is still unclear how exposure to VR modulates the neural control of balance, such as muscle activity and reflex excitability.

The primary objective of this study was to investigate visual feedback driven neural modulations in balance control across real (eyes open/closed; REO and REC) and virtual (low/high; VRL and VRH) conditions. In the real environment, standing with eyes closed served as a proxy to manipulate both arousal and visual feedback. In VR, this was accomplished by modifying platform height. We used mechanical (noisy tendon vibration – NTV) and electrical (soleus H-reflex) stimulation to discern global (spinal excitability) and fusimotor (muscle spindle-dependent) changes in reflex responses under these different visual feedback conditions. A secondary objective was to compare physiological (electrodermal activity) and psychological (fear, anxiety, and confidence questionnaires) indicators of stress when balancing at virtual height. Based on previous findings, it was predicted that: (i) physiological and psychosocial indicators of stress would be greatest in the VRH condition (Cleworth et al., 2012), (ii) similarly to real height platform studies, mechanically evoked stretch reflex amplitudes would increase (Horslen et al., 2013) under conditions of elevated postural threat from closing the eyes, and a virtual height platform, (iii) electrically evoked H-reflex amplitudes would not be altered by closing the eyes (Sibley et al., 2007), and (iv) H-reflex amplitudes will also be reduced (Llewellyn et al., 1990; McIlroy et al., 2003; Nafati et al., 2004; Sibley et al., 2007) or remain unchanged (Horslen et al., 2013), when standing on the edge of a virtual height platform.

## 2. Materials and methods

### 2.1. Experimental design

#### 2.1.1. Participants

A total of 20 healthy young adults (mean age: 23.3 ± 3.2 years; 10 females) participated in this study. Participants were free from musculoskeletal injury and any diagnosed neurological conditions. All participants had limited-to-no previous VR experience, and normal to corrected-normal vision with contact lenses, assessed via self-report. Participants requiring eyeglasses were excluded, as the eyeglasses could not fit underneath the VR headset. All participants provided written informed consent prior to taking part in this study. The University of Calgary’s Conjoint Health Research Ethics Board approved all experimental protocols.

#### 2.1.2. Procedure

**FIGURE 1:**
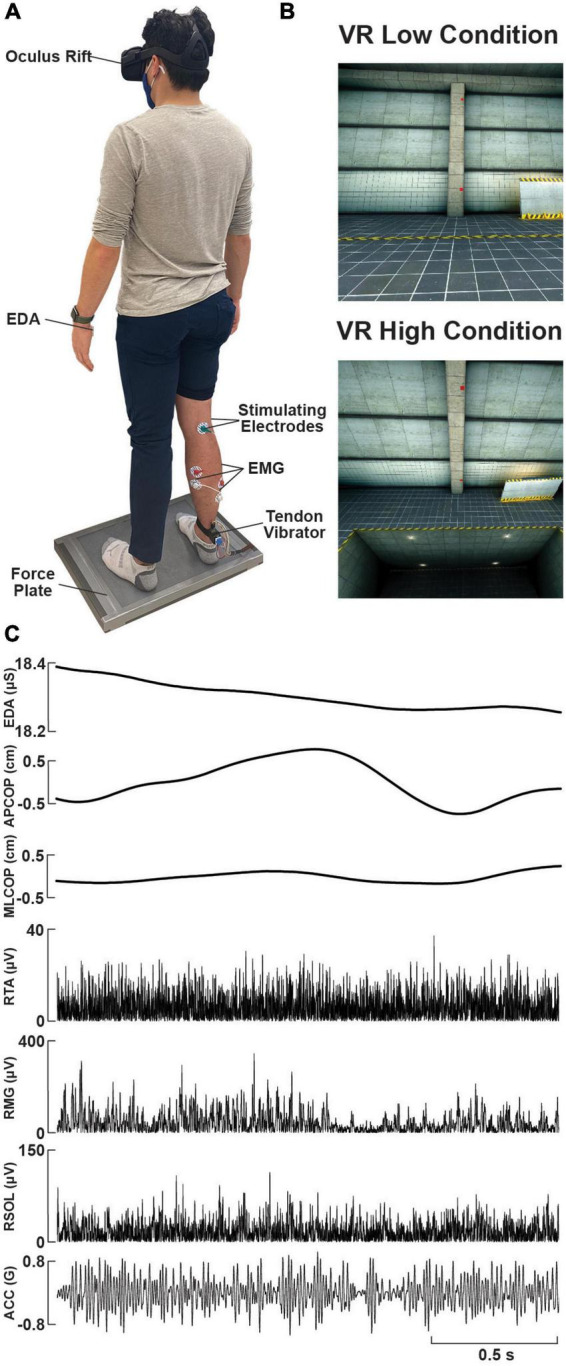
Experimental setup and raw data. **(A)** Photo of a participant standing on the force plate, instrumented with a VR headset, lower-limb surface electromyography (EMG) electrodes, tibial nerve electrical stimulation electrodes over the popliteal fossa, a custom-made wearable tendon vibrator over the right Achilles tendon, and electrodermal activity (EDA) electrodes on the left palm. **(B)** Sample visual environment displayed to participants within the ‘Low’ and ‘High’ (looking down) VR conditions. **(C)** Raw data traces of EDA, force plate (centre of pressure, COP, in the anterolateral, AP, or mediolateral, ML, planes), rectified EMG (right soleus, RSOL; right medial gastrocnemius, RMG; right tibialis anterior, RTA), and vibrator acceleration (ACC), during an exemplary trial.

Once written consent was obtained, participants were outfitted with EMG, EDA, and stimulating electrodes, as well as a custom-built wearable tendon vibrator (Figure 1). Prior to beginning the testing protocol, a one-time electrical stimulation recruitment curve protocol was performed to determine the maximum peak-to-peak amplitude and corresponding stimulus intensity values for the H-reflex (H-max), M-wave (M-max), and ½ H-max in the soleus muscle. The electrically evoked values vary between individuals; thus, the purpose for ascertaining this information was to determine an appropriate stimulation current for the testing protocol specific to each participant. To accomplish this, participants stood on a force plate with a fixed forward-facing gaze and arms relaxed at their sides while 1 ms square-wave pulses of electrical stimulation were delivered over the tibial nerve within the popliteal fossa. Stimulations were delivered at least 10 seconds apart to minimize risk of interference from presynaptic inhibition (Zehr, 2002; Palmieri et al., 2004). Stimulation current began at 5 mA and was subsequently increased in 2-5 mA increments until the desired physiological response was observed in the soleus. Once the H-wave amplitude began to descend with increasing current, the voltage supply was slowly dialed back during subsequent deliveries until the H-max was determined. To locate the M-max, 2-5 mA current increments were resumed until a plateau was observed in the rising M-wave. The H- and M-max were defined as the greatest peak-to-peak amplitude produced by the smallest amount of current. Finally, to locate ½ H-max, the stimulation current was reduced incrementally from H-max until the H-wave amplitude was equal to half of H-max. The current required to elicit ½ H-max peak-to-peak amplitude was used for the H-reflex assessment throughout the study. The ½ H-max targets a point near the middle of the ascending H-wave and is used to clearly differentiate between an increased/decreased response without ceiling or floor effects (Zehr and Stein, 1999; Sibley et al., 2007; Horslen et al., 2013). This recruitment curve protocol took approximately 5 min to complete, with 15-30 total electrical pulses delivered, and was followed by two minutes of seated rest prior to commencing the testing protocol.

During the subsequent testing protocol, participants experienced five test conditions while standing comfortably on the centre of the force plate with a fixed straight-forward gaze and arms at their sides. Each participant’s feet were separated by approximately 20 cm at the medial heel. The placement of the feet was kept consistent using tape markings on the force plate. Participants were instructed to stand relaxed throughout each of the five trials while NTV was used to probe T-reflexes (NTV-reflex) and tibial nerve stimulation was used to probe the H-reflex. During each condition, two minutes of NTV was applied to the right Achilles tendon either prior to or immediately following a series of five H-reflex pulses (separated by 10 s). The stimulus presentation order was interleaved across participants, such that half received NTV first and the other half received H-reflex pulses first, across all trials. In the real environment, participants completed one baseline trial with eyes open (REO), followed by another trial with eyes closed (REC). Participants were then introduced to the virtual environment. An Oculus Rift headset (Meta, CA, USA) displayed the VR environment coded through Vizard Enterprise 64-bit (WorldViz, CA, USA). The VR environment was a concrete warehouse with a small square platform flush with the floor, on which participants stood (Figure 1). A red square was positioned straight ahead on a concrete pillar and was used for a reference for participants to focus their gaze during trials. During an initial VR familiarization protocol, while standing on the force plate, participants were asked to: 1) describe their surroundings, 2) find three red cubes placed randomly around the room, and 3) turn their head and read the message on a screen located behind them (“Virtual Reality at the University of Calgary”). The familiarization protocol took 1-2 minutes to complete. Participants then completed one practice trial at ground level to mitigate potential first trial effects (Zaback et al., 2021). Two VR test trials proceeded with either the low (VRL) or high (VRH) condition performed first/second in randomized order. Half the participants received ‘VRL1, VRH, VRL2’ and the other half received ‘VRL1, VRL2, VRH’. We observed no significant effect of VR condition ordering on NTV (*F*(1,75) = 0.025; *p* = 0.875) and H-reflex (*F*(1,71) = 0.111; *p* = 0.739) responses, and therefore, we collapsed the data across the different orders. Prior to the VRH condition, the floor surrounding the platform was lowered seven meters and the platform was raised seven meters (net height change = 14 m) where another red square located at eye height was used for visual reference during the trial. Figure 1 depicts a visual representation of the scene in VRL and VRH conditions. Prior to each VR trial, participants completed a confidence questionnaire, and after each VR trial, participants completed a 16-item questionnaire addressing their perceived stability, fear, and anxiety during the trial (Adkin et al., 2002). Questions appeared on screen within the VR environment and participants were handed a controller to select their response on a numerical scale ranging from 0 to 100 (confidence, stability, and fear) or 1-10 (anxiety) scale. Each of the five condition trials lasted approximately 3 min including NTV and H-reflex assessment. Questionnaires following VR trials were completed in 3-5 min, and 60-s seated breaks were provided between all conditions to mitigate fatiguing effects.

### 2.2. Data collection

#### 2.2.1. Electrodermal activity

The galvanic skin conductance (i.e., sweat secretion) in response to each condition was monitored throughout the study by measuring electrodermal activity (EDA), which indicates the degree of sympathetic arousal (Boucsein et al., 2012; Horslen et al., 2013, 2017, 2018). For each condition, EDA during NTV and electrical stimulation was assessed separately rather than pooled together to account for any influence of stimulation method on arousal. Two surface electrodes (1-cm Ag/AgCl; MediTrace 133, Kendall, Technical products, Canada) were placed on the thenar eminence of the left hand (Cleworth et al., 2012). A Skin Conduction Unit (model 2502; CED Limited, England) recorded EDA with a sampling rate of 1 KHz.

#### 2.2.2. Psychosocial assessment

A series of questionnaires were delivered for conditions performed in VR to assess balance confidence, fear, stability, and anxiety (Smith et al., 1990; Adkin et al., 2002; Cleworth et al., 2012; Horslen et al., 2013, 2014, 2017, 2018). Individual questions appeared in the VR field of view, and participants were handed a controller to navigate to and select their appropriate response. Prior to each VR trial, participants rated their confidence in their ability to balance throughout the upcoming trial on a scale from 0% (no confidence) to 100% (complete confidence). Post-trial, participants rated their stability and fear on a scale of 0% (no fear; no instability) to 100% (completely fearful; complete stability), and a 16-item questionnaire assessed perceived anxiety using a nine-point scale ranging from 1 (I did not feel this at all) to 9 (I feel this extremely). These questionnaires have been demonstrated to have moderate to high reliability under height-induced postural threat in young adults (Hauck, 2011) and have been used in VR conditions (Nielsen et al., 2022). Scores from the anxiety questions were concatenated to achieve an overall measurement of anxiety.

#### 2.2.3. Electromyography

EMG was monitored to assess muscle activity throughout the study. Changes in muscle activity may be indirectly associated with changes in posture, fatigue, or arousal. Furthermore, changes in background EMG amplitude may influence other variables such as reflex amplitude and centre of pressure dependent variables. Prior to surface electrode placement, the skin overlaying the muscles of interest was shaved and cleaned with alcohol swabs. Two surface electrodes (1-cm Ag/AgCl; MediTrace 133, Kendall, Technical products, Canada) were applied to the skin, 2 cm centre-to-centre spacing, over the right soleus (SOL), medial gastrocnemius (MG), and tibialis anterior (TA). Electrodes were placed in line with muscle fiber orientation over the thickest region of the muscle belly (for MG and TA) or just distal and lateral to the gastrocnemius-soleus intersection for SOL. A reference electrode was placed over the lateral malleolus. EMG was sampled at 5 kHz and amplified with a gain of 2000 (NeuroLog NL844 pre-amplifier and NL820A isolated amplifier; Digitimer Ltd., England).

#### 2.2.4. Force plate

During testing, participants stood in the centre of a six-axis force plate (OR6-7; AMTI, USA). Ground reaction forces and moments, sampled at 1 kHz, were collected to monitor the center of pressure (COP) in the anterior-posterior and medial-lateral directions during all trials. See Figure 1 for a depiction of the experimental set up.

#### 2.2.5. Noisy tendon vibration

Mechanical stimulation of the Achilles tendon produced spindle-generated reflexes in the triceps surae muscles. A novel wearable device, secured to the posterior ankle using an elastic strap (2 cm above the calcaneus, directly between the medial and lateral malleolus) was used to deliver NTV (0 – 100 Hz) to the Achilles tendon. The elastic strap was secured in position and held at a snug, but comfortable tension prior to performing any study procedures and remained in place for the entire experiment. The device consisted of a custom-manufactured 3D printed housing (Figure 1) with onboard motor (Haptuator BM3C, Tactile Lab, Canada), amplifier (PAM8403), and accelerometer (ADXL354-CZ, Analog Devices Ltd., USA). Motor command signals were generated using custom LabVIEW software (National Instruments, USA) and output at 10 kHz from a real-time PXI system (PXIe-1062Q chassis; PXI-8105 embedded controller) with a multifunction data acquisition card (PXI-6289) and A/D board (BNC-2090). For the input signal to all subsequent NTV analyses, we computed acceleration magnitude as the square root of the sum of squared x, y, and z axis accelerations (in units of G). Using vibration magnitude is favorable in this case, as the wearable device vibrates in three-dimensions and we sought to fully capture this motion – this is unlike previous studies that used bulkier motors which moved only in one axis (e.g., Mildren et al., 2017).

#### 2.2.6. Electrical nerve stimulation

Electrical stimulation of the tibial nerve was required to generate H-reflexes in the triceps surae muscles. These reflexes are initiated along the peripheral nerve and therefore bypass the muscle spindle. Following previous studies, H-reflexes were used in conjunction with NTV-reflexes to assess site-specific adaptations (Davis et al., 2011, Horslen et al., 2013). Surface electrodes (1-cm Ag/AgCl; MediTrace 133, Kendall, Technical products Toronto, Ontario, Canada) were used for the anode and cathode. The anode was positioned in the centre of the popliteal crease in the popliteal fossa (Zehr, 2002), targeting the tibial nerve as it travels proximally and distally. The cathode was positioned 2 cm superior to the patella on the anterior thigh. A constant-current stimulator (STMISOLA; Biopac, USA) was used to deliver square-waveform electrical pulses (1 ms duration) using LabVIEW software (National Instruments, USA). The amperage (1-100 mA) of each stimuli was manually manipulated to achieve the stimulator output producing the desired physiological response.

### 2.3. Data processing

**FIGURE 2:**
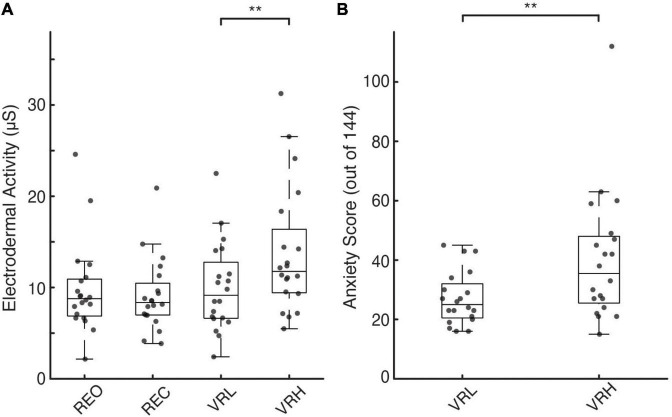
Physiological and perceptual indicators of postural threat-induced fear and anxiety. **(A,B)** Individual participant (gray circles) and box plots showing median and interquartile range for EDA under real **(A)** and virtual environments. **(B)** Individual and average participant questionnaire anxiety rating scores (out of a total of 0-144 points) under the different VR conditions. Asterisks denote significance at the *p* < 0.01** level.

**FIGURE 3:**
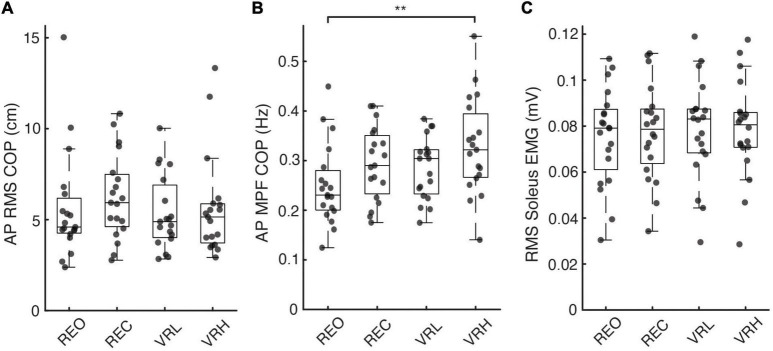
Postural sway and soleus muscle activity across real and virtual conditions. **(A)** Root-mean-squared (RMS) amplitude and **(B)** mean power frequency of anteroposterior (AP) centre of pressure (COP) excursions, as well as **(C)** RMS soleus muscle activity, across each visual condition. Asterisks denote significance at the *p* < 0.01** level.

Mean EDA, expressed in microSiemens (μS), was extracted during time windows encompassing the NTV and electrical stimulation periods. Force plate signals were digitally low pass filtered (dual-pass) at 10 Hz (4th order low-pass Butterworth filter) and COP was calculated from moments (Mx and My) and vertical force (Fz). The root mean squared (RMS) displacement and mean power frequency (MPF) of COP in the anteroposterior (AP) and mediolateral (ML) directions were calculated during each trial. EMG data were DC removed and full wave rectified for NTV analysis. Signals were digitally filtered at 1,000 Hz (dual-pass, 4th order, low-pass Butterworth filter). Background muscle activity was determined for the SOL, MG, and TA during H-reflex and NTV assessment by extracting the RMS amplitude during time windows encompassing the stimulation period plus one second on either end. For H-reflex assessment in the SOL, EMG signals were trigger-averaged to the stimulus delivery onset within a window of 10 ms preceding to 100 ms following tibial nerve stimulation. This resulted in a trace representing the mean response from the five H-waves evoked during each condition trial. Values were extracted for stimulus-triggered average peak-to-peak amplitude and latency from the stimulus to the first peak in the response wave (Figure 2). For NTV assessment in the SOL, coherence analysis was performed using the NeuroSpec2.0 software package (Rosenberg et al., 1989; Halliday et al., 1995) for MATLAB (Mathworks). This approach was adopted from prior studies investigating the time and frequency characteristics of stochastic stimuli-evoked physiological responses (Dakin et al., 2007, 2010, 2011; Mildren et al., 2017). Coherence values represent normative estimates of the frequency coupling strength between two signals (Mildren et al., 2017). Thus, coherence functions were used to assess the strength of the linear relationship between the input (NTV magnitude) and the output (SOL EMG) signals in the frequency domain. Coherence was estimated as the magnitude of the input-output signal cross spectra squared divided by the product of the input and output autospectra (Dakin et al., 2007; Mildren et al., 2017). Temporal characteristics of coherent frequencies were detected by using the inverse Fourier transform of the input-output signal cross spectra to compute cross-covariance (cumulant density), which was normalized by the product of the vector norms of the input and output signals. Resulting cross covariance values were normalized to a range of −1 to 1 (Dakin et al., 2010) and approximate the signal coupling strength in the time domain. Therefore, a positive correlation could be produced by either increased probe magnitude being coupled with an increase in EMG, or by decreased probe magnitude being coupled with a decrease in EMG. Independence of the two signals (input and output) are assumed under the constructed 95% confidence limits for coherence (positive threshold) and cross covariance (positive and negative thresholds) (Halliday et al., 1995). Values exceeding these limits are indicative of a significant linear relationship between the input (stimulus) and output (response) signals. Coherence spectra and cumulant densities were computed for each individual participant, as well as by pooling the data from each condition across all 20 participants (FFT windows = 1s; frequency resolution 1 Hz). Peak-to-peak and the timing of the cumulant density peak was extracted for statistical analysis. Additionally, we conducted pairwise Difference of Coherence tests (Amjad et al., 1997) to determine significant differences in pooled coherence spectra (see Figure 3).

### 2.4. Statistical analysis

Analysis was performed using custom MATLAB (MathWorks, MA, USA) and SPSS software (version 26.0; IBM SPSS Statistics Inc., Chicago, Il, USA) with α set to 5%. General linear mixed effects models were used to determine the main effect of visual condition on dependent variables. This statistical model provided comparisons between all conditions in real (REO and REC) and virtual (VRL and VRH) environments with pairwise estimates using sequential Bonferroni adjustments for multiple comparisons. Dependent variables in both real and VR environments included mean EDA (assessed independently during NTV and electrical stimulation), mean rectified background root mean squared (RMS) EMG in each muscle (SOL, MG, and TA), RMS and mean power frequency (MPF) of the anterior-posterior (AP) and medial-lateral (ML) COP, H-reflex peak-to-peak amplitude in the SOL, and NTV-reflex cumulant density peak-to-peak amplitude in the SOL. Pairwise difference of coherence tests compared coupling strength as a function of frequency for the identical planned comparisons. Subjective balance confidence, fear, stability, and anxiety data from questionnaires were assessed for VRL versus VRH using (one-tailed) paired *t*-tests. Mean EDA during each type of stimulus delivery (electrical vs. NTV) was compared for each condition using paired *t*-tests to determine if participant arousal was consistent during H-reflex and NTV-reflex assessment. Cohen’s *d* effect sizes were calculated, and magnitude of the effects may be interpreted as small (ES = 0.2), medium (ES = 0.5), or large (ES = 0.8) (Cohen, 1988).

## 3. Results

### 3.1. General results

Force plate analysis was conducted using data from 19 subjects; due to technical issues, these data were unavailable for one participant. Data from one participant was also excluded from the final H-Reflex analysis due to equipment malfunction. Of the remaining 19 participants, all had strong SOL muscle responses to H-reflex, while a weaker or absent response was observed in MG (18 responders) and TA (15 responders) muscles. Similarly, all 20 participants showed strong SOL and MG reflex responses (coherence) during NTV, while none definitively produced reflex responses in the TA. Since SOL consistently demonstrated the strongest response and was present in all individuals during H-reflex and NTV delivery, we focused the analysis exclusively on SOL. This approach is aligned with prior H-Reflex (Horslen et al., 2013; Young et al., 2018) and mechanical tendon stimulation (Horslen et al., 2013, 2018; Mildren et al., 2016, 2017) studies which focused on SOL for analysis.

### 3.2. Electrodermal activity

There were significant main effects for condition on EDA during both H-reflex assessment (*F*(3, 76) = 6.769; *p* < 0.001) and NTV (*F*(3, 76) = 7.003; *p* < 0.001). Pairwise contrasts, adjusted for multiple comparisons using the sequential Bonferroni correction, determined differences between specific conditions. As shown in Figure 2A, during NTV, significantly greater EDA was observed in the VRH condition compared to REO (41.6%; *t*(76) = 3.557; *p* = 0.003; CI: 1.045 – 7.08), REC (51.9%; *t*(76) = 4.126; *p* = 0.001; CI: 1.63 – 7.818), and VRL (38.4%; *t*(76) = 3.358; *p* = 0.005; CI: 0.912 – 6.756). There were no significant differences in EDA across stimulation method (electrical vs. NTV) for any condition. During electrical stimulation (H-reflex assessment), there was a similar increase in sympathetic arousal in the VRH condition compared to REO (39.6%; t(76) = 3.741; *p* = 0.002; CI: 1.324 – 7.692), REC (40.9%; t(76) = 3.826; *p* = 0.002; CI: 1.346 – 7.875), and VRL (35.1%; t(76) = 3.422; *p* = 0.004; CI: 1.041 – 7.207).

### 3.3. Psychosocial questionnaires

Questionnaires were administered prior to and following VR conditions only. We observed significant mean differences between the high and low conditions across all psychosocial measures. Compared to the VRL condition, participants reported significantly lower confidence in their ability to maintain balance prior to VRH (14.3%; *t*(19) = 2.63, *p* = 0.008, ES = 0.588). Similarly, during the VRH versus the VRL condition, participants felt more unstable (11.5%; *t*(19) = 2.87, *p* = 0.005, ES = 0.642) and more fearful of falling (17.3%; *t*(19) = −2.89, *p* = 0.005, ES = 0.646), while experiencing greater levels of anxiety (10.6%; *t*(319) = −7.3, *p* < 0.001, ES = 0.408; Figure 2B).

### 3.4. Background muscle activity

A general pattern of increased muscle activity emerged within the VR environment, most notably in VRH, in each muscle measured. *Soleus:* In SOL, there was a significant main effect for condition on background muscle activity (*F*(3, 76) = 2.832; *p* = 0.044). Pairwise contrasts, adjusted for multiple comparisons using the sequential Bonferroni correction, did not identify any significant difference between any conditions; however, non-significant trends for increased EMG were observed in the VR environment, with both VRL (3.8%; *t*(76) = 2.49; *p* = 0.075; CI: 0.000 – 0.004) and VRH (3.9%; *t*(76) = 2.557; *p* = 0.075; CI: 0.000 – 0.004) approaching significance compared to baseline (REO) (Figure 3). *Medial Gastrocnemius:* In MG, there was a significant main effect for condition on background muscle activity (*F*(3, 76) = 2.962; *p* = 0.037). Pairwise contrasts demonstrated an increase in EMG during VRH compared to baseline (REO) (10.4%; *t*(76) = 2.826; *p* = 0.036; CI: 0.000 – 0.007). *Tibialis Anterior:* Similarly, there was a significant main effect for condition on background muscle activity in TA (F(3, 76) = 3.151; p = 0.030). Pairwise contrasts demonstrated an increase in EMG during VRH compared to baseline REO (6.5%; *t*(76) = 2.953; *p* = 0.025; CI: 0.000 – 0.004).

### 3.5. Centre of pressure

Standing at virtual height impacted postural sway characteristics in the AP direction, indicated by a significant main effect for condition on AP COP MPF (*F*(3, 72) = 4.605; *p* = 0.005). Increases in AP COP MPF versus baseline (REO) were significant during the VRH condition only (31.1%; *t*(72) = 3.693, *p* = 0.003, CI: 0.021 – 0.135). There were no differences in COP RMS in the AP or ML direction, or in ML COP MPF, between any conditions. Figure 3 illustrates COP responses across conditions.

### 3.6. H-reflexes

**FIGURE 4:**
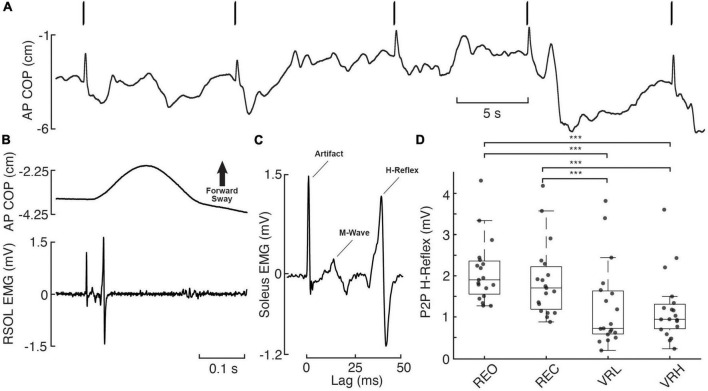
H-reflex responses across real and virtual visual conditions. **(A)** Anteroposterior (AP) centre of pressure (COP) during 5 consecutive electrical stimulation pulses (bars overtop), delivered to the tibial nerve at the popliteal fossa (S29, REO). A notable, forward-directed transient in AP COP postural sway resulting from rapid plantar flexion is observed in response to each electrical pulse. **(B)** Exemplary postural (AP COP) and muscular (RSOL EMG) response to electrical stimulation from a single pulse (initial pulse in panel **A**). **(C)** Stimulus-triggered average of the 5 consecutive pulses from this trial, with the RSOL stimulus artifact, M-Wave, and H-Reflex labeled. **(D)** Individual participant (gray dashed lines/diamonds) and average (black bars) peak-to-peak (P2P) H-reflex amplitudes. Asterisks denote significance at the *p* < 0.001*** level.

There was a significant main effect for condition on H-reflex P2P amplitude (*F*(72) = 13.829; *p* < 0.001). Significant pairwise contrasts demonstrate patterns of reduced H-reflex P2P amplitude within the virtual environment versus during real world conditions. Indeed, both VRL (40.8%; *t*(72) = −4.934, *p* < 0.001; CI: −1.315 – −0.397) and VRH (43.4%; t(72) = −5.247, *p* < 0.001; CI: −1.381 – −0.44) evoked smaller H-reflexes compared to baseline (REO). Similarly, H-reflex P2P amplitude was also reduced compared to REC during both VRL (34%; *t*(72) = −3.676, *p* = 0.001; CI: −1.063 – −0.212) and VRH (36.9%; *t*(72) = −3.988, *p* = 0.001; CI: −1.136 – −0.247). There was no difference in H-reflex peak-to-peak amplitude between VR height conditions (*t*(72) = −0.312, *p* = 0.756; CI: −0.4 – 0.292). A mean latency of 36 ms was observed, defined as the time between the stimulus delivery and the initial peak of the bi-phasic reflex wave (Figure 4).

### 3.7. Noisy tendon vibration reflexes

**FIGURE 5:**
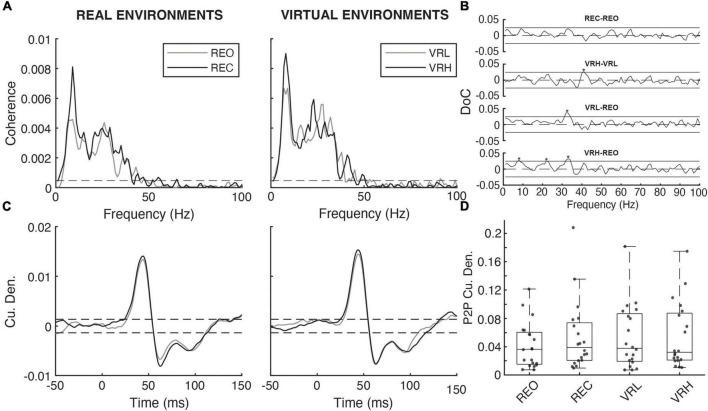
Noisy tendon vibration evoked responses across real and virtual conditions. **(A)** Pooled coherence spectral estimates (*N* = 20) under real (REO vs. REC) and virtual (VRL vs. VRH) visual environments. **(B)** Pairwise difference of coherence (DoC) tests comparing the pooled coherence spectra under real (REC-REO) and virtual (VRH-VRL) conditions. Comparisons between baseline (REO) and virtual (VRL-REO, VHR-REO) visual conditions are also shown. Solid horizontal black lines denote the 95% CI for each pairwise DoC test and values exceeding these lines (asterisks) indicate that, in all cases, NTV-muscular coherence was higher under conditions of greater postural threat. **(C)** Pooled cumulant density estimates for the relationship between vibration magnitude and rectified soleus activity under real and virtual visual environments. **(D)** Individual participant (gray dashed lines/diamonds) and average (black bars) peak-to-peak (P2P) cumulant density amplitudes.

A mean latency of 44.3 ms was observed, defined as the time between the stimulus delivery and the initial peak of the bi-phasic reflex wave. However, there were no significant condition effects on NTV-reflex P2P amplitudes. However, pairwise difference of coherence tests found that coherence between NTV magnitude and SOL muscle activity increased for a subset of frequency components (see Figure 5) for VRH vs. VRL, VRL vs. REO, and VRH vs. REO comparisons.

## 4. Discussion

### 4.1. Summary

The primary objective of this study was to investigate adaptations in arousal, postural control, and muscle stretch reflexes under different conditions of real and virtual visual feedback. We observed that mechanically evoked muscle stretch reflex amplitude remained unchanged across visual conditions, however, there were significant reductions in H-reflex amplitude in virtual reality. This phenomenon occurred not only when exposed to virtual height-induced threat, but also while standing at virtual ground level (VRL), a condition mimicking the REO baseline condition. Thus, merely altering visual input from a real setting to a virtual environment appears to alter the excitability of spinal reflex pathways. This lends support to existing literature suggesting that a specific adaptation may occur within the muscle stretch reflex circuity, such that, in VR, there is a global decrease in neuro-excitability at the level of the spine. Our results also suggest there may be a concomitant increase in muscle spindle sensitivity, which offsets this decrease in spinal excitability, keeping mechanically evoked muscle stretch reflexes unchanged (or even slightly increased) in amplitude. To appropriately address our primary research objective, this study also aimed to elicit physiological and psychological responses to postural threat using VR. A physiological arousal response to postural threat was observed, with significant EDA increases occurring with transition to VRH from baseline (REO), REC, and VRL conditions. Similarly, subjective anxiety and fear of falling increased, while balance confidence decreased in the VRH condition compared to VRL. Postural muscles of the lower limb also demonstrated increased co-contraction, which reached significance when transitioning to the VR height environment, likely explaining the increase in the mean power frequency of postural sway observed during this condition.

### 4.2. Psychosocial and physiological modulation

Significant changes in physiological and psychological responses in the virtual height condition compared to ground level are indicative of increased sympathetic arousal. Skin conductance (EDA) in the VRH condition was elevated compared to VRL, and concomitant reductions in perceived stability and balance confidence, as well as increased fear of falling were also documented. These findings support prior indications that VR height simulations can stimulate sympathetic arousal such as increased skin conductance, heart rate, and perceived anxiety and fear (Meehan et al., 2002; Cleworth et al., 2012) to a similar degree to real-world elevation. Additionally, no differences were observed in EDA between the real (REO) and virtual (VRL) baseline conditions. This indication that transitioning into VR does not increase participant arousal has been previously reported (Simeonov et al., 2008; Cleworth et al., 2012). The invariability in arousal between REO and VRL suggests that participants were not exposed to physiological stress during this transition, alluding that any associated changes in reflex excitability may be attributed to visual feedback rather than perceived threat. Overall, this study was successful in using VR to generate measurable physiological and psychosocial changes in sympathetic arousal.

### 4.3. Altered background muscle activity and postural sway

Here we observed a general pattern of increased muscle activity with the addition of virtual reality induced threat. Patterns of increased muscle activity in VR were significant during the height condition (VRH) compared to baseline (REO) for medial gastrocnemius and tibialis anterior. Increases in activity for these muscles at virtual height is consistent with the established increase in lower-limb co-contraction observed in real-world height induced postural threat. Simultaneously, postural sway frequency was also affected by VR; compared to baseline (REO), AP MPF increased in the VRH condition, indicative of a tensing response at the ankle joint. This likely represents a neural adaptation strategy to reduce instability (maintain balance) under conditions where the risk or consequence of a fall is heightened. A linear increase in COP frequency with height-induced threat has been previously reported (Adkin et al., 2000). In real height environments, increased muscular co-contraction alongside greater frequency of AP sway is observed.

Previous studies, which directly compared standing at ground level and height in both real and VR environments did not report significant differences in postural control between matched real and virtual conditions when standing on a firm surface (Simeonov et al., 2005; Cleworth et al., 2012), although Simeonov et al. (2005) did observe increased postural sway when standing on an unstable surface in VR versus a real environment for both ground level and height conditions. However, VR has been reported to increase postural instability during quiet stance on both firm and foam surfaces compared to similar real-world conditions; in fact, Horlings et al. (2009) observed that when compared to real-world quiet standing, the effects of VR on postural sway were similar to an eyes closed condition, consistent with the current study, which found no change in postural control between REC and VRL. It is possible that differences in technology or virtual visual environment account for the contradicting findings regarding postural control in VR. For example, the former studies created VR scenes that very closely matched their real-world laboratory (Simeonov et al., 2005; Cleworth et al., 2012), while Horlings et al. (2009) and the current study did not match the VR scene to the real-world eyes open condition. Participant VR experience may have also played a role in this discrepancy. While other studies do not report on this, participants entered the current study with very limited prior exposure to VR. It is possible that the novel nature of the VR experience could affect the results.

Other studies also did not report on changes in muscle activity in VR; however, the changes in postural control in this study are supported by the associated increase in muscular co-contraction described above. A review by Adkin and Carpenter (2018) identified eight studies which investigated height-induced postural and neurophysiological outcomes. A pattern emerged in the literature that is supported by the present research – each study reported evidence of increased COP frequency (Adkin and Carpenter, 2018). These previous studies also reported a reduction (6 studies) or no change (2 studies) in sway amplitude when threatened with height (Adkin and Carpenter, 2018). Recent research has substantiated these findings of altered balance control in response to standing in virtual elevation. Raffegeau et al. (2020) recorded postural deviations using inertial sensors while subjects were elevated in VR from ground level to 15 m height. The authors reported that AP postural responses (sway acceleration) were significantly greater at height, but only when standing parallel to the threat (i.e., on the edge of the platform compared to standing perpendicular to the edge).

### 4.4. Modulation in muscle stretch reflex responses

We observed that vibration evoked stretch reflexes remained unchanged, or marginally increased with virtual reality induced postural threat. Given that H-reflexes, and presumably, spinal excitability was diminished in virtual reality, our NTV results suggest that a compensatory neural mechanism is at play to maintain nearly constant mechanically evoked stretch responses. Indeed, in conditions where H-reflexes were reduced, NTV reflexes remained unchanged or slightly increased on average. This is consistent with previous reports of threat-induced modulations to postural control. Indeed, T-reflex amplification has been demonstrated to occur during static (Davis et al., 2011; Horslen et al., 2013) and dynamic reactive (Horslen et al., 2018) standing at height, as well as alternative sources of arousing stimuli (Bonnet et al., 1995; Both et al., 2003, 2005; Hjortskov et al., 2005; Kamibayashi et al., 2009).

As mentioned, an opposing trend to vibration reflex responses emerged for electrically evoked muscle stretch reflexes. H-reflex amplitudes were attenuated in VR conditions (VRL and VRH). No difference was observed in H-reflex amplitude between virtual height conditions in the present study. Interestingly, compared to REO, H-reflex amplitudes were reduced in the VRL condition, suggesting a more general effect of changing visual input from real to virtual, despite these conditions being seemingly similar. This demonstrates that the neural control of standing is altered in VR, even in the absence of any virtual height manipulation, which to our knowledge has not been reported previously (i.e., standing on ground level in the real- and virtual-world results are not equivalent). Reduced H-reflex amplitudes in response to threatening scenarios is consistent with previous reports. Subjects have exhibited weaker H-reflex amplitudes in response to an increased risk (Llewellyn et al., 1990; Horslen et al., 2013; Miranda et al., 2019) or consequence (Sibley et al., 2007; Horslen et al., 2013) of a fall, as well as during alternative scenarios stimulating sympathetic arousal (Kamibayashi et al., 2009; Tanaka, 2015). This suggests an inhibitory response at the spinal level that may be facilitated by descending commands from cortical or supraspinal structures (Llewellyn et al., 1990; Adkin et al., 2008). Tanaka et al. (2013) support this theory by using single-pulse transcortical stimulation to demonstrate increased corticospinal excitability to the internal oblique muscle during postural-challenging tasks at height. It is feasible that, when encountered with added sympathetic arousal or altered visual input, in general, the central nervous system downregulates spinal excitability to stifle excess motor outputs which may interfere with its ability to produce appropriate and efficient sensory-evoked responses to the stimuli. The net effect of this neural adaptation would be that mechanical reflex excitability remains unchanged, as observed in the present study.

### 4.5. Neural mechanisms for reflex modulation

We are under the assumption that both vibration and H-reflexes are short-latency responses using the same spinal circuitry except for initiation method and location. The H-reflex is initiated by stimulating the Ia afferent fibers directly (bypassing activation of muscle spindles), and therefore, its pathway is incomplete compared to its mechanically induced counterpart. Vibration reflex initiation occurs via muscle spindle stimulation resulting from a mechanical stretch applied to the muscle or tendon, encapsulating the entirety of the Ia afferent pathway. It is postulated, based on results of this study, that specific adaptations local to the muscle spindle may occur in the presence of virtual reality induced postural threat. This inference is consistent with prior research (Bonnet et al., 1995; Both et al., 2003, 2005; Hjortskov et al., 2005; Kamibayashi et al., 2009; Davis et al., 2011; Horslen et al., 2013, 2018) suggesting a heightened spindle-specific role in postural control in arousing situations. Furthermore, because significant H-reflex reduction signifies heightened inhibition at the spinal or supra-spinal level, the increase in spindle sensitivity alongside postural threat must be sufficient to overcome and exceed these inhibitions to register a net-positive increase in overall reflex. Therefore, it is likely that the muscle spindle response to threat is underestimated despite the significant enhancements observed here and in previous reports. The role of fusimotor control in posture and movement, and its specific adaptations when threats are perceived, demands further investigation by directly measuring spindle activity via single unit recordings with human microneurography.

Golgi Tendon Organ (GTO) inhibition may also play a role in NTV-reflex facilitation in threatening scenarios. Horslen et al. (2017) used electrical stimulation of the Achilles tendon to demonstrate Ib-inhibition in the triceps surae muscles during upright standing at both low and high height conditions compared to lying prone at ground level. Thus, it seems GTO-Ib reflexes are also dependent on task and threat perception, and their inhibition under these conditions may enable amplified short latency Ia-muscle spindle responses. Similar reductions in Ib inhibition have been previously demonstrated (i.e., Faist et al., 2006; Van Doornik et al., 2011) and are postulated to reflect a shift toward excitatory, rather than inhibitory reflexes during postural tasks compared to unloaded positions; thus, it seems reasonable to suggest this effect may be amplified as basic postural tasks (i.e., standing) advance to more threatening conditions (i.e., standing at height) with greater levels of muscle activation. However, axons of Ib afferents innervating GTOs are known to be smaller in diameter than Ia afferents supplying muscle spindles. Thus, their stimulation threshold requires a greater current – likely much higher than that delivered during this study, which used stimulation intensities in lower ranges to target muscle spindles specifically (Pierrot-Deseilligny and Burke, 2005). The concurrent roles of Ib-GTO inhibition and Ia-muscle spindle excitation in response to sympathetic arousal must be assessed further to confidently infer their contributions to postural tasks in threatening states.

### 4.6. Limitations and future considerations

One interpretation of our findings is that postural threat in VR increased fusimotor outflow, which maintained vibration reflex strength during a concomitant increase of spinal inhibition. A limitation of this research, however, is that we are inferring potential changes in fusimotor outflow and muscle spindle feedback indirectly using reflexive muscle activity as a proxy. As such, we cannot presently make firm conclusions regarding muscle spindle responses or potential mechanisms of fusimotor control. In future studies, these results should be bolstered by direct recordings from individual muscle spindles with human microneurography in threatening conditions. Even so, direct recordings from single fusimotor neurons are not feasible in humans; therefore, 𝝲MN activity will still need to be inferred from changes in muscle spindle firing, all while keeping αMN (muscle background activity) constant across conditions. This challenge of inferring fusimotor drive from muscle spindle activation is a well-appreciated in the literature (Burke, 2021; Dimitriou, 2021).

Another limitation to this study is the lack of a real-world high height condition. VR has previously been shown to be capable to eliciting similar responses to real-world conditions involving quiet standing at low and high heights (Cleworth et al., 2012). The current study aimed to build on this work by probing the state of neural circuitry under similar conditions. It would have been beneficial to directly compare these outcomes in real and virtual height conditions as well as in the real and virtual ground level conditions explored in this study. However, due to practical limitations, a real height condition was replaced with an eyes closed condition in this study. Nonetheless, closing the eyes enabled an interesting comparison to virtual environments, and has been used previously in studies involving real-world and VR visual environments (Horlings et al., 2009). Finally, comparisons between REO and VR conditions in current study would have been better controlled had the virtual environment been designed to match the real-world visual scene of the lab. Previous studies comparing real and virtual conditions have implemented near identical visual scenes within their virtual display (Simeonov et al., 2005; Cleworth et al., 2012), and this discrepancy may have played a role in some conflicting findings between studies.

Our data reinforces that VR can elicit sympathetic arousal and subjective fear responses with manipulations in virtual elevation. Future research should investigate how these outcomes scale in different, potentially even more threatening, virtual environments. Beyond basic neural mechanisms of balance control, our findings further suggest that VR may be useful for therapeutic purposes. For example, VR exposure therapy has been presented as a promising concept for managing phobias and anxieties, such as towards heights or flying, by gradually exposing patients to a negative stimulus (Rothbaum et al., 1997; Krijn et al., 2004; Bush, 2008; Albakri et al., 2022). There is already research in this area suggesting benefits in terms of fear of falling and fall risk in older adults (Rendon et al., 2012; Levy et al., 2016), and clinically in people with acrophobia (Coelho et al., 2009; Rimer et al., 2021), or anxiety disorders (Meyerbröker and Emmelkamp, 2011).

## Data availability statement

The raw data supporting the conclusions of this article will be made available by the authors, without undue reservation.

## Ethics statement

The studies involving human participants were reviewed and approved by University of Calgary’s Conjoint Health Research Ethics Board (CHREB). The patients/participants provided their written informed consent to participate in this study.

## Author contributions

DH wrote the first draft of the manuscript and performed the statistical analysis. JK and RP created the figures. DH, JK, and OD performed the data collection. All authors contributed to the study design, interpretation of the results, and editing of the final manuscript.
